# Supplementary material for: Content-rich biological network constructed by mining PubMed abstracts
Source: BMC Bioinformatics. 2004 Oct 8;5:147. doi: 10.1186/1471-2105-5-147 (PMC528731; doi:10.1186/1471-2105-5-147)
Supplement: Additional File 5 — The original Chilibot query results of the term "long-term potentiation (LTP)" and 22 other terms, limiting the latest references analyzed to the years 1990, 1995, 2000, and 2004. [file 1471-2105-5-147-S5.bz2 › chilibotAdditionalFile5/ltp2004/html/TAU_ACTIN.html]

 


 **TAU** and **ACTIN** 
  
Found 149 abstracts in PubMed,  **30 abstracts were retrieved and analyzed**.  


---

 Search Google  |
 PDF files only 
|  EDU domain only 

---

**Interactive relationship** (e.g. stimulation, inhibition, etc)

- **Actin**  disruption alters the localization of  **tau**  in the growth cones of cerebellar granule neurons.  Ref: 10893194 J Cell Sci, 2000
- The heterogeneous family of  **tau**  proteins interacts with microtubules,  **actin**  filaments, and intermediate filaments.  Ref: 10842324 J Cell Biochem, 2000
- Conversely, tubulin and assembled microtubules strongly inhibited  **tau**  interaction with  **actin**  in the solid phase system.  Ref: 11948687 J Cell Biochem, 2002
- The studies provide further evidence for the involvement of  **tau**  protein in modulating the interactions of microtubules and  **actin**  polymers in the organization of the cytsokeletal network.  Ref: 11948687 J Cell Biochem, 2002
- **Actin**  filaments decreased the extent of in vitro  **tau**  induced tubulin assembly.  Ref: 11948687 J Cell Biochem, 2002

**Parallel relationship** (e.g. studied together, co-existance, homology, etc.)

- In the present study, we determined the location of the dephosphorylated form of the microtubule associated protein  **tau**  dtau within the growth cones of newly forming axons and examined whether this localization was influenced by the  **actin**  cytoskeleton.  Ref: 10893194 J Cell Sci, 2000
- 0.05 but not for the cytoskeletal elements beta  **actin** , three repeat 3R  **tau** , and four repeat 4R  **tau** .  Ref: 12509874 J Comp Neurol, 2003
- Evidence indicating the presence of axonal mRNA in mammalian neurons has been limited to cytoskeletal components such as beta  **actin** ,  **tau** , and beta tublin, and is still a subject of controversial debate.  Ref: 12834963 J Biochem Biophys Methods, 2003
- Since cytoskeletal proteins play crucial roles in promoting neurite outgrowth, we examined the protein levels of some of these proteins that are associated with neurite outgrowth beta  **actin** , gamma  **actin** , alpha tubulin, MAP2 and  **tau** .  Ref: 11020545 Brain Res Dev Brain Res, 2000
- First, by measuring the effects of various ligands ATP, ADP, and P i on event lifetimes,  **tau**  on, in a laser trap, we determine the biochemical kinetics underlying the stepwise movement of an  **actin**  filament generated by a single myosin molecule.  Ref: 11916869 Biophys J, 2002
- To measure  **actin**  myosin protein breakdown, the 24 h excretion of N  **tau**  methylhistidine 3MH is used.  Ref: 12589703 Clin Sci (Lond), 2003
- In contrast, we observe that motion generation by an ensemble of myosin molecules is limited by ATP induced  **actin**  dissociationi.e., V varies as 1  **tau**  on at low ATP, but deviates from this relationship at high ATP.  Ref: 11916869 Biophys J, 2002
- Are  **tau**  and alpha synuclein filamentous inclusions in neurons and glia analogous to accumulation of myosin  **actin**  filaments in myofiber hypertrophy ? .  Ref: 12581622 Med Hypotheses, 2003
- Other cytoskeletal proteins, including  **actin** , microtubule associated protein, and  **tau** , are not influenced by chronic ECS administration.  Ref: 10611639 Synapse, 2000
- Tubulin,  **actin** , and  **tau**  protein interactions and the study of their macromolecular assemblies.  Ref: 11948687 J Cell Biochem, 2002
- **Tau**  protein association with tubulin immobilized in a solid phase support system was inhibited by  **actin**  monomer, and a higher inhibition was attained in the presence of preassembled  **actin**  filaments.  Ref: 11948687 J Cell Biochem, 2002
- Tissue sections were immunostained with vimentin, CD34, desmin, specific smooth muscle  **actin** , S100 protein, neuron specific enolase, PGP9.5, neurofilament, bcl 2 oncoprotein, synaptophysin, chromogranin A, c kit, and  **tau** .  Ref: 11727254 Hum Pathol, 2001
- Immunogold studies showed the association of  **tau**  along polymerized microtubules and  **actin**  filaments, even though a preferential localization of labeled  **tau**  with microtubules was revealed.  Ref: 11948687 J Cell Biochem, 2002
- In vitro reconstitution experiments of these cytoskeletal filaments using purified  **tau** , tubulin, and  **actin**  proteins were carried out.  Ref: 11948687 J Cell Biochem, 2002
- Cytoskeleton structural proteins like  **actin** , tubulin, L neurofilaments and  **tau**  protein, axon specific antibodies, other neuronal or glial proteins like S 10 4 3 3 and glial fibrillary acid protein, neuronal specific enolase, and nitric oxide and its metabolites are some of the putative markers that deserve further investigation and validation.  Ref: 14652789 Neurol Sci, 2003
- Degenerative disruption of neuronal networks would appear to constitute a pathogenetic series of mechanisms that would adequately account for the effects of neurodegeneration in its full complexity clinically and pathologically beyond any consideration of the neuron as the basic unit of involvement.In this sense, neurodegeneration as reflected in terms of neurofibrillary tangles and Lewy bodies might actually be a process of integral  **tau**  opathy and synucleinopathy that in probably multiple different ways in the individual patient contributes to a disruption of neuronal interactions as an integral neuronal network.In terms therefore of aggregation of neurofilaments or of synuclein filaments it might be valid to consider simple biophysical phenomena of inclusion body formation in neurons directly analogous to myosin  **actin**  accumulation in myofibers in an overall common system of neuronal and myofiber biology and pathobiology.  Ref: 12581622 Med Hypotheses, 2003
